# One-piece versus two-piece ceramic dental implants

**DOI:** 10.1038/s41415-024-7123-3

**Published:** 2024-03-08

**Authors:** Curd Bollen, Gagik Hakobayan, Martin Jörgens

**Affiliations:** 4141534847001 Professor, Ulster University, College of Medicine and Dentistry, Birmingham, UK; 4141534847002 Professor and Head of Department of Oral and Maxillofacial Surgery, Yerevan State Medical University after Mkhitar Heratsi, Yerevan, Armenia; 4141534847003 Professor, University of Sevilla, Spain; MUHAS University, Dar es Salaam, Tanzania

## Abstract

In this narrative review, a structured comparison between one-piece and two-piece zirconia dental implants is highlighted. Ceramic dental implants have long ceased to be hype; on the contrary, they can offer a significant addition to the daily dental implant practice. Not only do their favourable aesthetics play a significant role, but their ability to work completely metal-free is of added value, particularly for patients with a proven allergy for Grade 5 titanium. Furthermore, the fact that peri-implantitis seems to appear only incidentally is an important supporting argument for their use as well. Whereas the original design of zirconia implants was formerly always of a one-piece/one-phase structure (the monobloc design), nowadays, two-piece/two-phase designs (the so-called hybrid concept) are also widely utilised to restore missing teeth. Both concepts have advantages and disadvantages, scientifically as well as clinically.

For this paper, relevant articles from the recent scientific literature were selected from PubMed. The aim was to identify and summarise what has previously been published on one-piece versus two-piece ceramic implants. This article will compare the benefits and drawbacks of one-piece versus two-piece ceramic implants based on clinical- (design, different sizes, surgical protocol, prosthetics), scientific- (loading and eventual complications) and patient-related (costs and long-time perspectives) criteria.

## Key points

- Ceramic implants are a valuable alternative for titanium implants.
- One-piece zirconia implants can be restored as natural teeth.
- Abutments of two-piece ceramic implants can be attached with teflon, titanium, gold or ceramic screws.
- Zirconia implants offers the solution for patients requesting a complete metal-free restoration.

## Introduction

In the dental implant community, there is still a lot of discussion on the place of ceramic dental implants in the rehabilitation of (partial) edentulous patients. A majority still considers zirconia implants as a transient phenomenon, whereas others consider it as the ultimate breakthrough in implant dentistry.^1^

Ceramic dental implants are a relatively new type of dental implant made from the ceramic material zirconia (zirconium dioxide [Zro_2_]).^2^ In the past, ceramic implants were predominantly made of aluminium oxide (Al_2_O_3_), which was a far too brittle material for oral rehabilitation, leading to multiple implant fractures, causing a widespread rejection in their application, and to a global stigmatisation of ceramic dental implants.^3^

Recently, ceramic dental implants are becoming increasingly popular again due to their aesthetic appeal and biocompatibility.^4^^,^^5^ Unlike traditional titanium implants, ceramic implants have a whitish colour, making them virtually indistinguishable from natural teeth, especially when the patient presents with a thin gingival biotype.^6^ In such cases, the hint of grey titanium in combination with a high smile line is an aesthetic letdown.

Additionally, ceramic implants are hypoallergenic, making them a suitable option for patients with metal allergies.^7^ Titanium allergy can be detected in dental implant patients, even though its estimated prevalence is quite low (0.6%). A higher risk of positive allergic reaction was found in patients showing post-op allergy compatible responses (allergic symptoms after implant placement or unexplained implant failures).^8^

These implants also have a lower thermal conductivity compared to metal implants, which can reduce sensitivity and discomfort in the mouth, often experienced as unpleasant by the patient.^9^

Whereas ceramic implants are still relatively new, research has shown promising results in terms of their long-term success rates and durability.

**Fig. 1:**
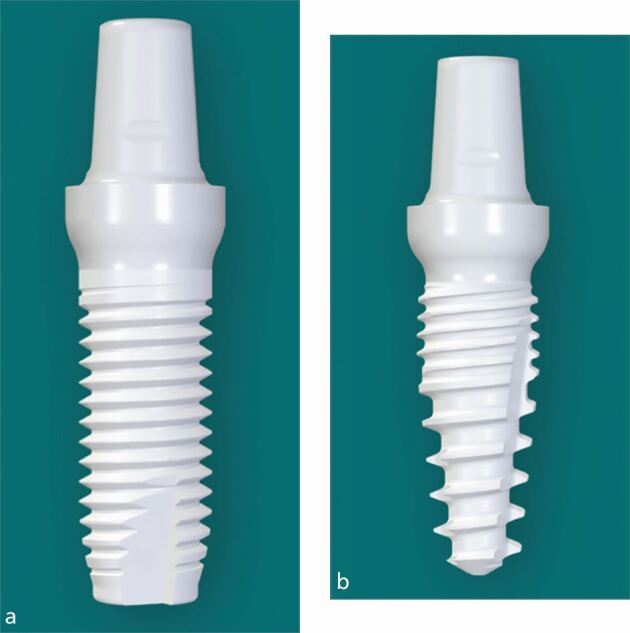
a, b) One-piece ceramic dental implants (Z-Systems: Z5m & Z5mt, Oesingen, Switserland)

The choice between a one-piece/one-phase implant versus a two-piece/two-phase implant is a more recent phenomenon. At the early days of ceramic dental implants, all these implants were produced as a monobloc, that is, an implant with an integrated abutment (Fig. 1).^10^

On one-piece/one-phase implants, there are more studies published since they have already been on the market much longer. In the seventies, Sammy Sandhaus and Thomas Driskell were publishing groundbreaking work. Both proved separately the great opportunities of working with ceramic one-piece implants.^11^^,^^12^

**Fig. 2:**
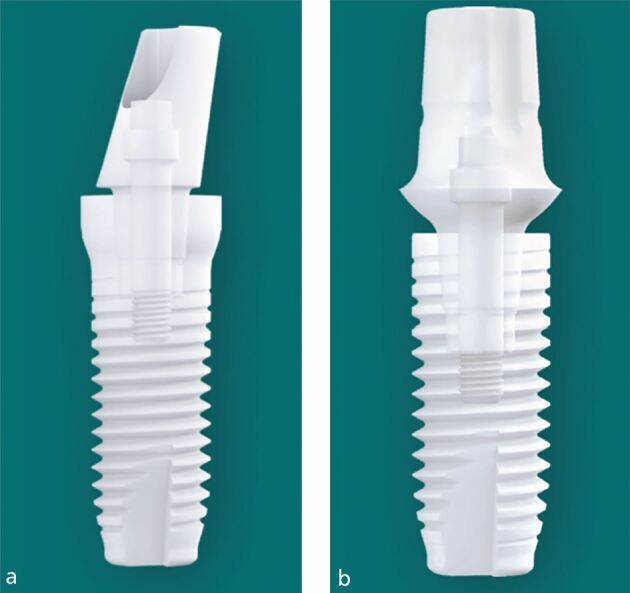
a, b) Two-piece ceramic dental implants (Z-Systems: Z5-TL & Z5-BL, Oesingen, Switserland)

Only more recently have two-piece/two-phase ceramic implants entered the dental implant market (Fig. 2, Table 1).

**Table 1:** Detailed overview of the two-piece/two-phase ceramic implants and their components

Brand	Product	Cemented	Screwed	Screw
Z-Systems	Z5-BL/Z5-TL	No	Yes	Ceramic or titanium
Zeramex	XT/P6	No	Yes	Carbon
Nobel Biocare	NobelPearl	No	Yes	Carbon
Straumann	Pure	No	Yes	Titanium
Zircon Medical	Patent	Yes	No	-
WITAR	AWI	No	Yes	Direct*
Neodent	Zi	No	Yes	Titanium
Camlog	Ceralog	No	Yes	Titanium or gold
SDS Swiss Dental Solutions	Bright/Value	Yes	Yes	Peek or titanium
TAV Dental	W	No	Yes	Titanium
Key: * = Ceramic abutment directly screwed into the implant (no additional screw)				

Due to their later release on the market, these two-piece/two-phase implants have less scientific data available and the existing data span up to ten years.^13^^,^^14^ Although the medium-term results are excellent after 5-6 years, the German Society for Implantology made a warning in their recent S3 guideline.^15^^,^^16^ Thiem and co-workers confirm the feasible use of one-piece zirconia implants as an addendum/alternative to titanium implants. However, no conclusion regarding the application of two-piece ceramic implant systems can be drawn based on the existing data. So, they suggest recommending these implants only after the patient has been informed in detail about the lack of long-term clinical data.

## Criteria

Based on eight different criteria, the differences, and advantages/disadvantages between one-piece and two-piece ceramic dental implants will be discussed.

### Design

With a one-piece implant, the implant and the abutment are fused to one simple monobloc. Therefore, there can't be any bacterial leakage between the implant and the abutment because there is no joint as with the two-piece implants, where there is always a gap detected between the implant and the abutment.^17^ This means furthermore that the temporary or final crown must ultimately be cemented on top of the implant. There is a wide range of these implants commercially available (Table 2).

**Table 2:** Overview of one-piece and two-piece ceramic dental implants

One-piece ceramic implants	Two-piece ceramic implants
Z-Systems: Z5m/Z5m(t)	Z-Systems: Z5-BL/Z5-TL
Straumann: Pure Monotype	Straumann: Pure
Camlog: Ceralog Monobloc	Camlog: Ceralog Hexalobe
Zircon Medical: Patent one-piece	Zircon Medical: Patent two-piece
SDS Swiss Dental Solutions: Bright	SDS Swiss Dental Solutions: Bright/Value
TAV Dental: W-1	TAV Dental: W-2
WITAR: AWI one-piece	WITAR: AWI two-piece
ZiBone	ZiBone
Medical Instinct: BoneTrust	Neodent: Zi
Fair Implant: Fair White	Zeramex: XT/P6
CeraRoot	Nobel Biocare: NobelPearl
Tree-Oss Ceramic	SIC invent: SICwhite
Bredent: WhiteSKY	

**Fig. 3:**
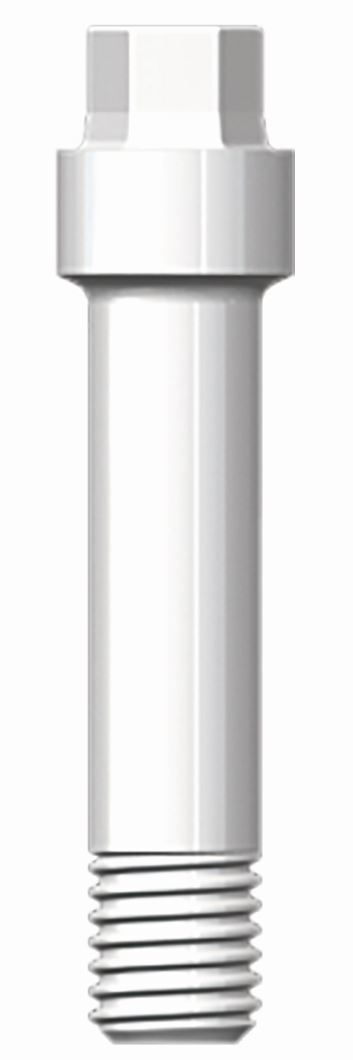
Ceramic abutment screw (Z-Systems, Oesingen, Switserland)

The more complex two-piece implants consist of two or three parts: the implant body itself, the abutment, and the abutment retention screw. In case of a cementable abutment, there is, of course, no abutment screw. The retention screw can be fabricated out of titanium, gold, carbon, or zirconia. If the patient wants a complete metal-free restoration, an internal ceramic abutment screw is required (Fig. 3).

It is important to follow the manufacturers‘ instructions for applying correct torque on these screws: titanium screw is 25 Ncm; carbon screw is 25 Ncm; zirconia screw is 12 Ncm; and gold screw is 15 Ncm. Currently, there are only a limited number of two-piece implants on the dental market (Table 2).

Considering the design, there are parallel and tapered implants available. Most of the implants are not self-tapping. Therefore, in almost all situations, bone tapping is advised before implant installation.

**Fig. 4:**
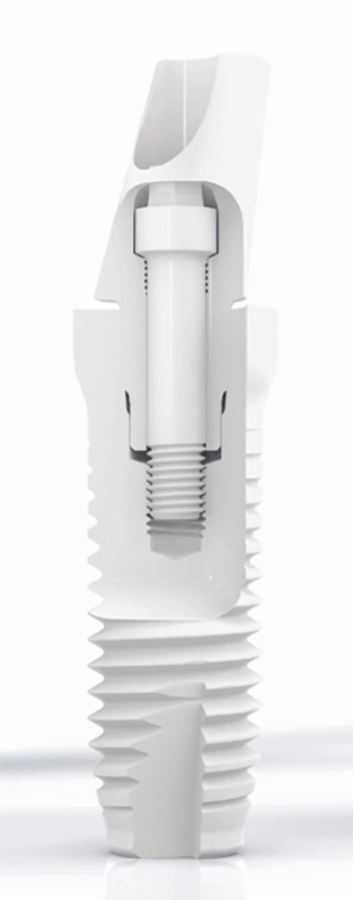
Conical internal connection with anti-rotational apex (BL, Z-Systems, Oesingen, Switserland)

For the two-piece implants, there is a large variety of internal connections. Not every connection offers the same stability. An internal conical connection with anti-rotational apex is preferred (Fig. 4).

### Surgery

The first-stage surgical procedure for both implant types is identical, although for one-piece implants, a flapless approach can be appropriate. The flapless technique for one-piece implants shows, however, statistically significantly more bone loss, which might be indicative for future problems.^18^

Only in a two-stage approach for two-piece implants is a second surgery necessary, connecting the healing abutment to the implant. Healing abutments are mostly made from PEEK (polyetheretherketone) or PEKK (polyetherketoneketone).

Because it is not always allowed to prep one-piece zirconia implants (always carefully look at the manufacturer recommendations), their immediate correct surgical positioning is of utmost importance.^19^

Therefore, it can be advantageous to initially use guided surgery for these procedures, helping to avoid incorrect inclination of the abutment component of the implant.^20^ For two-piece implants this problem is less significant since most commercial brands offer angulated or preparable abutments in their portfolio.

Whether one-piece or two-piece implants are installed, low drilling speeds should always be applied, assuredly when ceramic implant drills are applied. Drills made of ceramics do not conduct the warmth, leading to overheating of the bone in the osteotomy.^21^ The latter doesn't lead to implant failure but induces significant crestal bone loss during healing and a final lower percentage of bone-to-implant contact.^22^ These drilling speeds start around 800 rpm for the first drills, reducing to 400 rpm for the last drills. The advised tapping for D1-D2 (and D3) bone should be performed at 15 rpm.^23^ It is of utmost importance to check the individual recommendations of the manufacturer before using the respective drill sequences.

### Loading

Since for ceramic implants bone tapping is almost always utilised, the primary stability of these implants is often insufficient for direct loading.^24^ Therefore, delayed, or late loading are mostly recommended for two-piece implants. Moreover, in the anterior aesthetic area, a two-phase technique can help to improve the gingival aesthetic outcome, as shown by Suchetha and co-workers.^25^

**Fig. 5:**
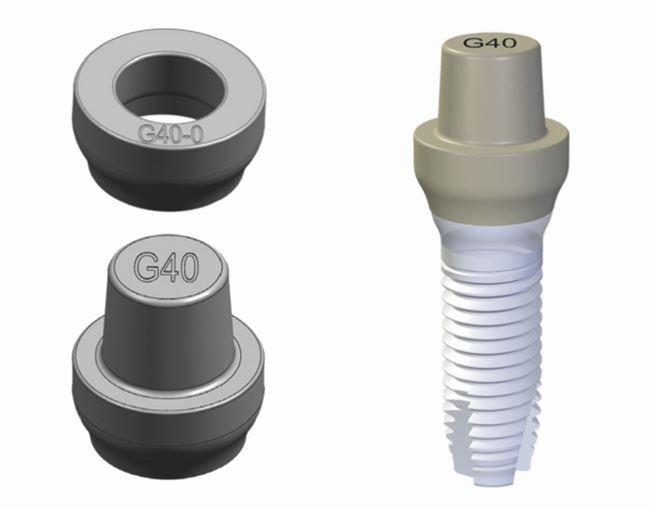
PEEK protection caps for one-piece implant (Z-Systems Z5m, Oesingen, Switserland)

One-piece implants are, due to their design, directly loaded. To reduce these immediate loading forces, most brands offer silicone or PEEK protection caps to place over the abutment after installing the implant. These shock absorbers also protect against gingival overgrowth during the required healing time (Fig. 5).

### Prosthetics

The prosthetic procedure of a one-piece implant is almost completely identical to the prosthetic process for natural teeth. Both analogue and digital impressions are possible. Due the high affinity of the soft tissue towards zirconia, often, excess gingiva must be controlled by retraction cords or (diode) laser.^26^ Implant analogues are not really required in this method.

For two-piece implants, the procedures are identical as for titanium two-piece implants: analogue or digital impressions; open or closed tray. Different brand-related scan bodies are available and here an implant analogue is always needed for the further laboratory handling. It is still of the highest importance to use the original components, delivered by the respective manufacturers, since printing of these individual components does not yet offer the same accuracy.^27^

### Sizes

The offer in diameters and lengths is rather limited for one-piece compared to two-piece ceramic implants, but there is no structural difference in portfolio between both types of implants: sizes are similar. Table 3 shows the ranges in diameters and lengths of the actual most commonly used ceramic dental implants.

**Table 3:** Range in diameters and lengths of different commercially available ceramic dental implant systems

Brand	Range of diameters	Range of lengths
Z-Systems	3.6-5 mm	8-12 mm
Zeramex	3.5-5.5 mm	8-14 mm
Straumann	3.3-4.8 mm	8-14 mm
Nobel Biocare	3.5-5.5 mm	8-14 mm
Camlog	4 mm	8-12 mm
Zircon Medical	4.1-5 mm	7-13 mm
SDS Swiss Dental Solutions	3.2-7 mm	6-17 mm
TAV Dental	3.6-4.8 mm	8-14 mm
Bredent	3.5-4.5 mm	8-16 mm
ZiBone	3.6-5 mm	8-14.5 mm
Tree-Oss	3.7-4.3 mm	10-13 mm
CeraRoot	3.5-6.5 mm	8-14 mm
Neodent	3.75-4.3 mm	10-13 mm
WITAR	3.9-6 mm	8-14 mm
Fair Implant	3.5-5 mm	8-13 mm
Medical Instinct	4-5 mm	10-13 mm
SIC invent	3.5-5.5 mm	8-14 mm

The available diameter ranges from 3.3 (Straumann) to 7 mm (SDS). The lengths range from 6 mm (SDS) to 16 mm (Bredent). The average diameter is 4.2 mm and the average length is 10.8 mm. With these sizes, almost all indications are properly covered.

### Costs

The use of one-piece implants is relatively less expensive since there is only need for a full ceramic crown that can be cemented on top of the implant-abutment complex. For two-piece implants there is always the need for extra components: ceramic abutments and abutment retention screws. These extra components mean not only an extra cost in their purchase from the manufacturer, but also an extra cost in the laboratory handling, making the final cost of a two-piece ceramic implant substantially higher. The latter is the case for all brands who offer both one-piece and two-piece zirconia implants.

### Complications

The main complication for oral implants is the absence or lack of osseointegration. With the actual ceramic materials, the success rates of zirconia implants are comparable with those of titanium implants. After all, zirconia and titanium implants show a similar soft and hard tissue integration capability. Titanium, however, tends to demonstrate an accelerated initial osseointegration compared to zirconia. It is meanwhile also clear that zirconia implants do not show better clinical results as titanium implants.^28^^,^^29^ So both systems seem to have comparable clinical outcomes.

With one-piece implants, the cementation of the crown can cause cement rests that can remain present subgingivally. These toxic cement rests can easily induce peri-implantitis.^30^ Therefore, the meticulous removal of all excess cement after cementation of the crown is of utmost importance.^31^

As mentioned before, the incorrect positioning (that is, inclination) of a one-piece implant that may not be ground post-operatively is a major problem. Here, the only solution is explantation. Two-piece ceramic implants can offer different complications. Abutment screw loosening and abutment screw fracture are the main problems.^32^ Therefore, it is essential to apply the exact prescribed torque value when installing the abutment or the crown. The more components used, the higher the risk for complications.

As far as scientific literature is concerned, there seems to be less peri-implantitis around ceramic in comparison with titanium implants.^33^^,^^34^ A peer explanation on this phenomenon is still awaited. Although there is no scientific literature available yet, clinically there seems no difference in peri-implantitis rates between one-piece and two-piece implants.^35^

### Patient perspective

This is probably an underestimated and neglected factor in daily clinical decision-making. Patients prefer minimal invasive therapy, minimal morbidity, minimal number of appointments and minimal costs. When comparing one-piece and two-piece implants, it is obvious that patients will prefer their therapy with one-piece implants because this concept offers the most advantages for them.

Moreover, the recent S3 guideline on ceramic implants by the German Society of Implantology advises all practitioners to warn their patients that there is still insufficient scientific data to support the unlimited use of two-piece ceramic dental implants.^16^ The latter should therefore in fact always be consented to before applying two-piece implants in practice.

## Conclusions

In implant dentistry, it can be stated that one-piece implants offer the same prognosis as two-piece implants. Moreover, recent studies indicate clearly that one-piece as well as two-piece ceramic implants show excellent clinical results. However, two-piece ceramic dental implants do not offer sufficient long-term scientific substantiation yet to support their overall use in daily practice. Therefore, an extended informed consent should always be offered to patients receiving therapy with two-piece zirconia implants.

The use of two-piece zirconia implants will increase since they offer much more versatility than one-piece implants. This higher versatility will unfortunately result in a rise of the costs for the practitioners and consequently for the patients. Future randomised controlled trials will have to confirm the promising results of two-piece zirconia implants.
